# The Role of Cardiovascular Magnetic Resonance Imaging in the Evaluation of Hypertrophic Cardiomyopathy

**DOI:** 10.3390/diagnostics12020314

**Published:** 2022-01-26

**Authors:** Sanjay Sivalokanathan

**Affiliations:** 1 Internal Medicine, Pennsylvania Hospital, University of Pennsylvania Health System, Philadelphia, PA 19107, USA; sanjay.sivalokanathan@pennmedicine.upenn.edu; 2 Cardiovascular Clinical Academic Group, St. George’s University of London and St George’s University Hospitals NHS Foundation Trust, London SW17 0RE, UK

**Keywords:** cardiac MRI, hypertrophic cardiomyopathy, sudden cardiac death

## Abstract

Hypertrophic cardiomyopathy (HCM) is the most common inherited cardiac disorder, affecting 1 out of 500 adults globally. It is a widely heterogeneous disorder characterized by a range of phenotypic expressions, and is most often identified by non-invasive imaging that includes echocardiography and cardiovascular magnetic resonance imaging (CMR). Within the last two decades, cardiac magnetic resonance imaging (MRI) has emerged as the defining tool for the characterization and prognostication of cardiomyopathies. With a higher image quality, spatial resolution, and the identification of morphological variants of HCM, CMR has become the gold standard imaging modality in the assessment of HCM. Moreover, it has been crucial in its management, as well as adding prognostic information that clinical history nor other imaging modalities may not provide. This literature review addresses the role and current applications of CMR, its capacity in evaluating HCM, and its limitations.

## 1. Introduction

Hypertrophic cardiomyopathy (HCM) is an inherited (60–70%) cardiomyopathy, affecting 0.2–0.5% of the general population across the globe, and has been the focus of extensive research over the last five decades [1]. It is a highly heterogeneous disorder, attributed to the myriad of mutations involving the cardiac sarcomere proteins. Common mutations include genes encoding myosin heavy chain 7 (MYH7) and myosin-binding protein c (MYBPC3). It is characterized by left ventricular hypertrophy (LVH), with or without signs of left ventricular (LV) outflow tract obstruction, diastolic dysfunction, myocardial ischemia, and mitral regurgitation. Although the annual mortality rate is 1%, with the estimated survival rates being 98% at 5 years and 95% at 10 years [2], a small subset of patients is subject to adverse complications that includes sudden cardiac death (SCD), heart failure, and tachyarrhythmias [3]. It is also the most common identified cause of SCD in athletes in North America [4]. It is evident that cardiovascular magnetic resonance (CMR) imaging is key towards the recognition of HCM, as well as aiding in management of the disorder. CMR can provide three-dimensional (3D) imaging, with high spatial and temporal resolution that does not involve any ionizing radiation. Furthermore, CMR is well suited to describe the various expressions of HCM, with an important feature being the ability to delineate the etiology of left ventricular hypertrophy from other causes that includes athlete’s heart, hypertension, valvular disease, and infiltrative diseases. This literature review discusses the role of CMR in HCM, its current applications, and its limitations.

## 2. Materials and Methods

We performed a review of the literature on PubMed and Scopus, focusing on cardiovascular magnetic resonance imaging and hypertrophic cardiomyopathy (Supplementary Table S1, Appendix A). Reviews, meta-analyses, prospective, retrospective, interventional, and observational studies were included in our search. The exclusion criteria included conference abstracts, case studies, or articles where there was no association between CMR and HCM. Key search terms included “hypertrophic cardiomyopathy”, “HCM”, “hypertrophic”, “inherited cardiomyopathy”, “left ventricular hypertrophy”, and “LVH” in combination with “cardiovascular magnetic resonance imaging”, “CMR”, “cardiac imaging”, “cardiac magnetic resonance imaging”, “perfusion imaging”, and “cardiac MRI”.

## 3. Results

### 3.1. Definition of HCM

HCM is a disorder characterized by inappropriate myocardial hypertrophy that occurs in the absence of any systemic or detectable causes. Histopathologically, it is defined by myocyte disarray, microvascular dysfunction, and interstitial and perivascular fibrosis. It was first described in the late 1950s, and whilst the initial diagnosis of HCM was made by pathologists, advancement in imaging has facilitated prompt diagnosis, risk-stratification, and earlier utilization of interventions to curb cardiovascular complications [5,6]. HCM is diagnosed when one or more LV myocardial segments, at end-diastole, is ≥15 mm, or the septal to lateral wall thickness ratio is higher than 1.3 in a non-dilated LV, in the absence of a loading condition [7]. The three main types of HCM, typically identified through CMR, include asymmetric, concentric, and apical. Asymmetric or septal HCM accounts for two-thirds of the spectrum, which may lead to systolic anterior motion (SAM) of the mitral valve, and subsequent left ventricular outflow tract (LVOT) obstruction [8]. The apical variant accounts for less than 10% of HCM patients [9]. Complications of HCM include sudden death, heart failure, and arrhythmias. In HCM patients with heart failure, the annual mortality is ten-fold higher, with the increase in risk being attributed to progressive pump failure and SCD [10].

### 3.2. Cardiovascular Magnetic Resonance vs. Echocardiography

Whilst conventional echocardiography is considered the first line imaging modality for the clinical diagnosis of HCM, it is operator dependent and often limited by its acoustic windows and lack of tissue characterization [11]. Conversely, CMR allows a more comprehensive and detailed evaluation of hypertrophy, with accurate measurements of wall thickness, chamber size, and distribution of hypertrophy (Table 1). It is also capable of evaluating coronary flow reserve, contractility, and tissue perfusion, permitting the reproducible assessment of cardiac abnormalities [12]. One of the significant advantages of CMR includes detailed anatomical assessment in different planes, providing a three-dimensional representation of anatomy [13]. Thus, contouring the epicardial and endocardial borders allows the calculation of quantitative parameters, including consistent measurements of atrial and ventricular function and volumes. A typical CMR examination involves electrocardiographic (ECG) synchronized cine acquisitions, at a strength of 1.5 or 3T [14]. There are three main techniques used in clinical CMR, which include spin echo imaging, gradient echo imaging, and flow velocity encoding [13]. Steady-state free precession (SSFP), related to gradient echo imaging, involves generating high temporal and spatial resolution cine images, which are important for functional assessment [15]. This offers a greater distinction between muscle and blood, with different ratios of T2 and T1, which is suitable for cardiac imaging and more beneficial for segmentation algorithms.

Furthermore, with contrast, there have been additional capabilities of CMR that include late gadolinium enhancement (LGE) assessment and myocardial T1 and T2 mapping. CMR also has the capability of distinguishing phenotypic expression, segmental LVH, and the identification of fibrosis and structural abnormalities. For instance, assessing the extent of LVH of the lateral or basal anterior LV wall and the LV apex, or the assessment of LV outflow and/or midflow obstruction, is better evaluated by CMR [16]. Moreover, CMR provides a clear demarcation between the blood pool and the myocardium, which enables a more accurate calculation of LV volumes and mass [17].

In addition, the advantage of CMR over echocardiography includes better characterization of mitral valve abnormalities as well as identifying the mechanisms of LV outflow obstruction [17]. The discrepancy might be vast between the two imaging modalities, in which the difference may be as high as 70% in classifying massive LVH [18]. Other useful information CMR provides includes the identification and quantification of right ventricular (RV) hypertrophy, microvascular dysfunction, and assessment of diastolic function. LVOT obstruction is another important manifestation of HCM that is better evaluated by CMR, whereby the identification and grading in severity of the obstruction is cardinal for offering invasive treatment that includes myomectomy or alcohol septal ablation. In this sense, CMR has the added advantage of locating the site of flow obstruction, and any specific anomalies that contribute to it as well.

### 3.3. Late Gadolinium Enhancement

**Figure 1:**
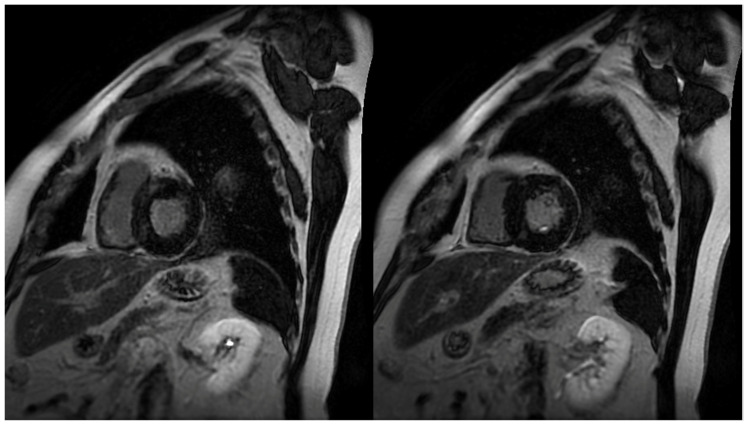
Short axis images demonstrating patchy late gadolinium enhancement (LGE) in hypertrophic cardiomyopathy (HCM) patients. Common patterns identified in HCM patients include localization in segments with greatest **left** ventricular hypertrophy (LVH), and **right** ventricular (RV) insertion points.

The identification and quantification of LGE is a valuable feature of CMR, changing the paradigm in how ischemic and non-ischemic myocardial diseases are assessed (Figure 1). It represents myocardial fibrosis and is a predictor of both a higher mortality rate and the progression towards heart failure amongst HCM patients [19]. The mean reported prevalence of LGE is 65%, but may be present in up to 86% of HCM patients [7,20]. Typical patterns include localization to the mid-wall, located in segments with the greatest LVH, and at RV insertion points. Thus, peculiar patterns of LGE, during the assessment of LVH, can be attributed to other diagnoses that may include, but are not limited to, Anderson–Fabry disease or cardiac amyloidosis (CA). The distinction between the location of LGE includes the type of fibrosis, whereby intramural mid-wall LGE is considered a marker for replacement fibrosis, whilst LGE at RV insertion points suggests interstitial fibrosis [21]. Replacement fibrosis increases diastolic dysfunction and ventricular stiffness [10], and plays a role in the progression of heart failure. It is well acknowledged that the presence and extent of LGE is associated with disease severity, which includes the extent of LV thickness, remodeling, and dysfunction [17,22]. It has also been suggested that it may act as a substrate for both arrhythmias and heart failure [23]. More importantly, LGE is an independent predictor of sudden death, whereby if >15% occupies the LV wall, it results in adverse remodeling and a twofold increase in the risk of sudden death [24]. The presence of LGE is thus proposed to be a substrate, accounting for a greater risk, in ventricular tachyarrhythmias [23,25].

LGE patterns in HCM patients do vary greatly, with a wide range of locations and distributions being described. The most common pattern, affecting 30% of patients, is described as patchy, affecting the septum and free LV wall [26]. Other locations include the isolated involvement of the apex, septum, lateral wall, and the right ventricular insertion points. Often, extensive LGE is located in the walls of apical aneurysms associated with HCM [27]. LGE is, however, frequently absent in HCM patients without LVH, suggesting the association between pathological hypertrophy and LGE enhancement [27]. In addition, the presence of LGE has not shown a clear relationship with a reduction in ejection fraction (EF), but has been implicated in myocardial stiffness, regional wall motion abnormalities, and magnitude of LVOT obstruction [26,28,29]. Nonetheless, it dictates follow up, as those with significant LGE do require closer monitoring given the risk of progression in systolic dysfunction [30,31].

### 3.4. T1 and T2 Mapping

Early myocardial structural changes in cardiomyopathies are often elusive and indistinguishable. LGE was initially viewed as the gold standard for assessment of fibrosis in HCM. However, with the development of more sensitive techniques, the European Society of Cardiology (ESC) has described a paradigm shift towards the role of parametric mapping in assessing myocardial integrity [32,33]. Therefore, T1, T2, and T2* mapping has become a routine part of the CMR exam, with changes in values showing early remodeling changes. T1 mapping provides the assessment of the total extent of expanded extracellular space [34,35], and is thus emerging as a tool for the quantification of subtle fibrosis [36,37]. Longitudinal relaxation time, which depends on T1, varies between tissues and pathological conditions. Whilst LGE signifies focal fibrosis and myocardial scar, native and post-contrast T1 mapping detects diffuse interstitial fibrosis [38]. Moreover, subtle LGE enhancement may not be easily appreciable, and there may be errors in myocardial nulling during LGE assessment. Therefore, native and post-contrast T1 mapping has become a significant tool for prognostication in challenging cases. Extracellular volume (ECV), derived from T1 mapping, measures the proportion of extracellular space between myocytes. In HCM patients, both native T1 and ECV have been demonstrated to be prolonged, which correlates with the hypertrophied segments, suggesting interstitial fibrosis [39]. More importantly, these findings were associated with an increased susceptibility to ventricular arrhythmias, and thus sudden death [40]. The values are also utilized to differentiate from other causes of cardiac hypertrophy that include amyloidosis [41], hypertensive cardiomyopathy [42], and Fabry’s disease [43].

T2 mapping, however, demonstrates myocardial or interstitial water content with variations in signal intensity being useful in differentiating between different cardiac diseases [44]. In HCM, higher T2 values are reported, with the differences being accounted for on a structural level, which are purported to reflect myocyte degeneration, disarray, and replacement fibrosis [45]. In addition, it frequently corresponds with LGE, which may explain the process of initial dysfunction in HCM and the subsequent progression to fibrosis [46,47]. Another technique is T2* blood oxygen level-dependent (BOLD) MRI, which evaluates myocardial oxygenation. In HCM patients, the values are reduced, reflecting areas of reduced perfusion, and correlate with T1 mapping, showing a temporal association between fibrosis and silent ischemia [2]. Similar to the presence of LGE, abnormalities with T2* values were also associated with ventricular arrhythmias [48].

### 3.5. Strain Measurement

HCM patients often exhibit a hyperdynamic LV, and therefore the assessment of true LV function is of paramount importance. Similar to echocardiography, strain measurements include measurements in radial, circumferential, and longitudinal directions, reflecting both global and regional myocardial function. With a higher spatial resolution offered by CMR, it is argued that it displays a more superior and sensitive estimation of LV function than echocardiography. Here, LV strain is assessed using myocardial tissue tagging, where tagged CMR can assess regional myocardial mechanics at different time-points during the cardiac cycle [49,50]. Several studies have reported HCM patients, despite a seemingly normal LV ejection fraction, as having globally reduced strain, which is associated with both increased mortality and heart failure events [51,52]. The dysfunction of the LV in HCM is silent, and given myocardial contraction is heterogeneous, the different markers of strain assessment are useful in delineating the type of contractile dysfunction. For instance, the hypertrophied segments often exhibit reduced early diastolic strain rates [53]. Such aggravation in myocardial integrity correlates biochemically, with elevated NT-proBNP and troponin T, and is also reflected by an increased likelihood of ventricular arrhythmias [18,54]. Furthermore, the impairment in strain often correlates with the presence of LGE, and its role has been extended to the assessment of the right ventricle and left atrium [52,55]. Given that approximately 1 in 5 HCM patients are affected by atrial fibrillation (AF) [56], the evaluation of left atrial strain is becoming more essential, as it may predict the risk of AF, but also provide the clinician with a cause for closer monitoring [54,55,57,58].

A proponent of CMR is speckle tracking imaging. Speckle tracking echocardiography (STE) involves tracking patterns provided by speckles, which are stable acoustic markers [59]. These are sites of positive interference with the ultrasound wave, and depend on the orientation of the scattering sites and surrounding ultrasound field. The orientation of the myocardial architecture changes within each cardiac cycle, which is reflected by changes in speckles. An additional advantage of STE includes having a lower signal-to-noise ratio than CMR. However, the tissue tracking technology is based on estimations of tissue displacement, and thus may not be representative of true myocardial motion, may be affected by blood motion, and is often operator dependent [59].

### 3.6. Perfusion CMR

Microvascular dysfunction (MVD) is another common feature thought to be responsible for ischemia-mediated myocyte death in HCM, which is argued to result in replacement fibrosis and adverse LV remodeling. This is corroborated in postmortem studies that reveal the presence of ischemic damage in HCM hearts, without any significant coronary artery disease, that varies from acute to chronic fibrotic changes [60,61]; other noteworthy changes include arteriolar dysplasia, without significant plaque formation [10]. In order to assess the presence and impact of MVD, indirect measures such as myocardial perfusion reserve (MPR), derived by the ratio of myocardial blood flow (MBF) during maximal coronary vasodilation to MBF at rest, provides the means of estimating MVD. Adenosine stress perfusion MRI is highly sensitive and is the method that may assess such myocardial perfusion defects in HCM patients with suspected MVD [62,63,64,65]. Perfusion defects are present in up to half of HCM patients, and is a predictor of clinical decline [10]. As a result, it has been viewed as a target, albeit unsuccessful thus far, for the prevention of disease progression [62]. A frequent finding using this technique was stress perfusion defects along hypertrophied segments, often correlating with the degree of maximal wall thickness in HCM patients [61]. Such findings were also associated with ventricular arrhythmias, the presence of apical aneurysms, and increased LV mass index [61]. It was also common for HCM patients (30%) to demonstrate perfusion defects at rest, which often correlates with the presence of LGE and a reduction in contractile function [66,67]. Thus, it is suspected that the presence of such findings are risk factors for HCM patients to develop adverse cardiovascular effects that include sudden cardiac death.

More importantly, several studies have also shown a correlation between MVD and LGE burden [68]. However, these perfusion defects may manifest themselves prior to the development of LGE [69,70,71]. This was complemented by a recent study showing genotype-positive, phenotype-negative carriers of HCM in demonstrating both global and segmental perfusion defects, prior to the development of LGE [72,73], therefore suggesting that microvascular abnormalities may precede the development of cardiac hypertrophy and fibrosis.

### 3.7. Microstructural Dysfunction

Microstructural changes are argued to precede macroscopic abnormalities in HCM, and in patients with normal wall thickness, and without any discernable scar, it is important to devise a technique that may appreciate these abnormalities and thus provide the viable markers necessary for the early detection of disease, screening of family members, and for prognostication. Cardiac diffusion tensor imaging (cDTI) is a method that allows the characterization of the myocardial microstructure and may provide such a solution. There are several parameters of measurement, including mean diffusivity (MD), fractional anisotropy (FA), voxel-wise helix angle (HA), and secondary eigenvector angle (E2A). MD measures the magnitude of diffusion in a given voxel, and is said to be increased in areas of interstitial fibrosis in HCM patients [74]. FA measures the directional variability of diffusion in a given voxel, and is a measure of myocyte disarray, with low values suggesting adverse clinical outcomes, such as ventricular arrhythmias [74,75,76]. HA describes myocyte orientation, and E2A reflects orientations of laminar sheetlets [57]. Studies have demonstrated that HCM patients have increased MD, E2A, and reduced FA in comparison to normal subjects [76,77]. Higher MD and E2A was seen to correlate with areas of scarring, fibrosis, increased ECV, and LV wall thickness, whereas a reduction in FA was a marker for areas of fibrosis [77]. Whilst this may be an investigative tool, and its clinical applicability is still being explored, such in vivo biomarkers may provide an additional means of risk stratifying HCM.

### 3.8. Flow Imaging

The assessment of blood flow is important to the clinical evaluation of cardiovascular disease. Phase-contrast imaging allows the visualization and quantification of flow, and is widely used in cardiac imaging for the functional assessment of regional blood flow in the heart, across the valves, and in great vessels [78,79,80]. It involves taking advantage of the direct relationship between blood flow velocity and phase of the MR signal, and through correction sequences and subtraction of unwanted signals, velocity encoded images are generated [79,81]. Important basic functions of phase-contrast MRI include the estimation of cardiac output and evaluation of diastolic dysfunction. The measurement of diastolic dysfunction may be done by measuring the flow across the mitral valve, yielding information on early (E) and late or atrial (A) ventricular filling patterns [13,81].

One of the uses of phase-contrast MRI includes assessing obstructive HCM, a common disease subtype, characterized by thickening of the interventricular septum and associated with systolic anterior motion (SAM) of the mitral valve, in which the leaflets, during mid-systole, contact or near contact the septum. A component of HCM that is also often not highlighted as much in literature is aortic stiffness, with the LVOT-to-ascending aorta (AAo) diameter ratio correlating with the outflow gradient [82]. The degree of LVOT obstruction is necessary to evaluate, as it is associated with structural disadvantages that include diastolic dysfunction and reduced LV compliance, leading to adverse cardiovascular events such as heart failure, stroke, and SCD [83]. The hemodynamic assessment of the LVOT gradient in HCM using echocardiography is limited, and with recent developments, four-dimensional (4D) flow MRI, which can visualize 3D flow patterns, may provide a more comprehensive assessment of the LVOT pressure gradient and ascending aortic flow [15]. The findings include altered pressure gradients and abnormal flow patterns in the LVOT and AAo, demonstrating that as the gradient increases, it leads to worsening outcomes [15,84].

## 4. Discussion

HCM is a widely heterogeneous disorder that is associated with sudden cardiac death and heart failure, but often presents without any symptoms. Thus, its variability in presentation makes it challenging to risk stratify the disorder and delineate its disease course. Reassuringly, with CMR, there has been greater confidence in evaluating HCM and its associated disease severity. There are several applications of CMR that describe HCM well, both at a subclinical and molecular level. Methods include LGE assessment, strain analysis, and T1/T2 mapping. Whilst LGE detects replacement fibrosis, parametric mapping can detect interstitial fibrosis and changes prior to focal scar formation. For instance, T1 and T2 remodeling occurs even in normal appearing myocardial segments, suggesting that tissue remodeling precedes functional and morphological changes in HCM patients [85]. There has been an overwhelming number of studies and evidence in the use of LGE, and it is becoming a recognized arbitrator in assessing the risk of SCD. For instance, if >15% LGE occupies the LV wall, it dictates whether an implantable cardioverter-defibrillator (ICD) should be considered, as well as the length of follow-up. Undoubtedly, one of the advantages of CMR over echocardiography is its better visualization and assessment of the LV wall and thickness. For instance, the identification of apical hypertrophy and aneurysms may only be identified through CMR. The other use of CMR includes preprocedural planning, which allows the evaluation and follow-up of remodeling post interventions such as septal myectomy or ablation. The influence and potential of CMR is extensive, with new and emerging applications that include the use of cDTI, hyperpolarized ^13^C MRI, and CMR spectroscopy involving assessment of myocardial dysfunction and energy status at a cellular level [86]. Moreover, as the incidence of unexplained LVH increases, novel quantitative markers have been developed. For instance, myocardial contraction fraction (MCF), calculated by dividing the LV stroke volume by LV myocardial volume, discriminates HCM from CA and hypertensive heart disease [87].

On the other hand, it should be noted that there are several limitations in the utility of CMR. Much of the functional analyses require manual editing, which may result in over- or underestimation. Other common barriers include longer examination times, reimbursement issues, and lack of availability and expertise. Furthermore, the interpretation of LGE in HCM patients can be challenging and may be overexaggerated. For instance, the LGE signal may differ from one study to another, and is influenced by technical parameters that include the threshold set to differentiate normal from fibrotic myocardium. However, this has been resolved with the use of semi-automated analysis for quantification. Here, using signal intensity in normal myocardium as a reference, five or six standard deviations from the normal myocardial intensity seem to correlate the best with visual assessment when identifying LGE [9]. Within multiparametric mapping, factors such as co-morbidities, age, and gender play a role with the values obtained. More importantly, centers use different vendors, mapping sequences, and field strength, which makes it difficult at times to incorporate complex protocols and apply them to the general HCM population.

## 5. Conclusions

HCM is an inherited cardiomyopathy with a wide range of clinical presentations and long-term sequelae. Whilst many patients have an indolent course, a considerable number of patients are at an increased risk of SCD, heart failure, or tachyarrhythmias. Thus, imaging has come to play a central role in the diagnosis and prognostication of HCM. CMR is a widely employed tool that aids in the diagnosis and clinical management of HCM patients. Its capability in providing unique information on cardiac function, morphology, and tissue characterization has surpassed its own expectations, and with continued technological advancement, it is only a matter of time before pre-existing techniques are refined and newer methods are devised to even further characterize HCM.

## Figures and Tables

**Table 1:** There are several advantages of cardiovascular magnetic resonance imaging (CMR) that include functional evaluation, morphological visualization, and risk stratification. There is also the added benefit of differentiating HCM from other similar processes that result in cardiac hypertrophy, such as amyloidosis, athlete’s heart, and hypertensive heart disease.

Advantages of Cardiovascular Magnetic Resonance Imaging
Identification of HCM phenotypes
Accurate quantification of maximal wall thickness
Assessment of co-existing valvulopathies
Volume analysis and quantification
Perfusion and strain analysis
Assessment of LVOT and cause
Offers differential diagnosis
Risk stratification through identification of fibrosis

**Figure A1:**
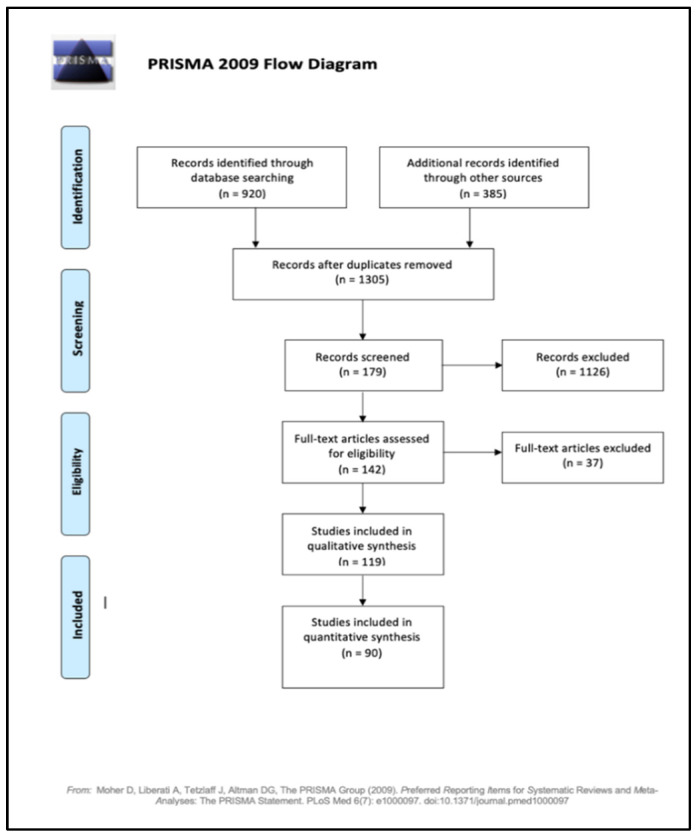
PRISMA flow diagram describing the selection of studies for review [88].

## Data Availability

Data sharing is not applicable to this article.

## Supplementary Materials

The following supporting information can be downloaded at: https://www.mdpi.com/review/10.3390/12020314. Supplementary Table S1: PRISMA 2020 Checklist.

## References

[1] Maron B.J., Gardin J.M., Flack J.M., Gidding S.S., Kurosaki T.T., Bild D.E. (1995). Prevalence of hypertrophic cardiomyopathy in a general population of young adults. Echocardiographic analysis of 4111 subjects in the CARDIA Study. Coron. Artery Risk Dev. Young Adults. Circ..

[2] Ando K., Nagao M., Watanabe E., Sakai A., Suzuki A., Nakao R., Ishizaki U., Sakai S., Hagiwara N. (2020). Association between myocardial hypoxia and fibrosis in hypertrophic cardiomyopathy: Analysis by T2* BOLD and T1 mapping MRI. Eur. Radiol..

[3] Cheng S., Fang M., Cui C., Chen X., Yin G., Prasad S.K., Dong D., Tian J., Zhao S. (2018). LGE-CMR-derived texture features reflect poor prognosis in hypertrophic cardiomyopathy patients with systolic dysfunction: Preliminary results. Eur. Radiol..

[4] Malhotra A., Sivalokanathan S. (2021). The transatlantic evolution in understanding sudden cardiac death in athletes. Trends Cardiovasc. Med..

[5] Rowin E.J., Maron B.J., Carrick R.T., Patel P.P., Koethe B., Wells S., Maron M.S. (2020). Outcomes in Patients with Hypertrophic Cardiomyopathy and Left Ventricular Systolic Dysfunction. J. Am. Coll. Cardiol..

[6] Brock R. (1957). Functional obstruction of the left ventricle; acquired aortic subvalvar stenosis. Guy’s Hosp. Rep..

[7] Noureldin R.A., Liu S., Nacif M.S., Judge D.P., Halushka M.K., Abraham T.P., Ho C., Bluemke D.A. (2012). The diagnosis of hypertrophic cardiomyopathy by cardiovascular magnetic resonance. J. Cardiovasc. Magn. Reson..

[8] Maron M.S., Olivotto I., Harrigan C., Appelbaum E., Gibson C.M., Lesser J.R., Haas T.S., Udelson J.E., Manning W.J., Maron B.J. (2011). Mitral valve abnormalities identified by cardiovascular magnetic resonance represent a primary phenotypic expression of hypertrophic cardiomyopathy. Circulation.

[9] Bogaert J., Olivotto I. (2014). MR Imaging in Hypertrophic Cardiomyopathy: From Magnet to Bedside. Radiology.

[10] Raphael C.E., Mitchell F., Kanaganayagam G.S., Liew A.C., Di Pietro E., Vieira M.S., Kanapeckaite L., Newsome S., Gregson J., Owen R. (2021). Cardiovascular magnetic resonance predictors of heart failure in hypertrophic cardiomyopathy: The role of myocardial replacement fibrosis and the microcirculation. J. Cardiovasc. Magn. Reson. Off. J. Soc. Cardiovasc. Magn. Reson..

[11] Grajewski K.G., Stojanovska J., Ibrahim E.H., Sayyouh M., Attili A. (2020). Left Ventricular Hypertrophy: Evaluation with Cardiac MRI. Curr. Probl. Diagn. Radiol..

[12] Waterhouse D.F., Ismail T.F., Prasad S.K., Wilson M.G., O’Hanlon R. (2012). Imaging focal and interstitial fibrosis with cardiovascular magnetic resonance in athletes with left ventricular hypertrophy: Implications for sporting participation. Br. J. Sports Med..

[13] Markl M., Kilner P.J., Ebbers T. (2011). Comprehensive 4D velocity mapping of the heart and great vessels by cardiovascular magnetic resonance. J. Cardiovasc. Magn. Reson. Off. J. Soc. Cardiovasc. Magn. Reson..

[14] Scheffler K., Lehnhardt S. (2003). Principles and applications of balanced SSFP techniques. Eur. Radiol..

[15] Allen B.D., Choudhury L., Barker A.J., van Ooij P., Collins J.D., Bonow R.O., Carr J.C., Markl M. (2015). Three-dimensional haemodynamics in patients with obstructive and non-obstructive hypertrophic cardiomyopathy assessed by cardiac magnetic resonance. Eur. Heart J. Cardiovasc. Imaging.

[16] Brenes J.C., Doltra A., Prat S. (2018). Cardiac magnetic resonance imaging in the evaluation of patients with hypertrophic cardiomyopathy. Glob. Cardiol. Sci. Pract..

[17] Suinesiaputra A., Bluemke D.A., Cowan B.R., Friedrich M.G., Kramer C.M., Kwong R., Plein S., Schulz-Menger J., Westenberg J.J., Young A.A. (2015). Quantification of LV function and mass by cardiovascular magnetic resonance: Multi-center variability and consensus contours. J. Cardiovasc. Magn. Reson. Off. J. Soc. Cardiovasc. Magn. Reson..

[18] Pu C., Fei J., Lv S., Wu Y., He C., Guo D., Mabombo P.U., Chooah O., Hu H. (2021). Global Circumferential Strain by Cardiac Magnetic Resonance Tissue Tracking Associated with Ventricular Arrhythmias in Hypertrophic Cardiomyopathy Patients. Front. Cardiovasc. Med..

[19] Green J.J., Berger J.S., Kramer C.M., Salerno M. (2012). Prognostic value of late gadolinium enhancement in clinical outcomes for hypertrophic cardiomyopathy. JACC Cardiovasc. Imaging.

[20] O’Hanlon R., Grasso A., Roughton M., Moon J.C., Clark S., Wage R., Webb J., Kulkarni M., Dawson D., Sulaibeekh L. (2010). Prognostic significance of myocardial fibrosis in hypertrophic cardiomyopathy. J. Am. Coll. Cardiol..

[21] Quarta G., Aquaro G.D., Pedrotti P., Pontone G., Dellegrottaglie S., Iacovoni A., Brambilla P., Pradella S., Todiere G., Rigo F. (2018). Cardiovascular magnetic resonance imaging in hypertrophic cardiomyopathy: The importance of clinical context. Eur. Heart J. Cardiovasc. Imaging.

[22] Liu J., Zhao S., Yu S., Wu G., Wang D., Liu L., Song J., Zhu Y., Kang L., Wang J. (2021). Patterns of Replacement Fibrosis in Hypertrophic Cardiomyopathy. Radiology.

[23] Elliott P.M., Gimeno J.R., Thaman R., Shah J., Ward D., Dickie S., Tome Esteban M.T., McKenna W.J. (2006). Historical trends in reported survival rates in patients with hypertrophic cardiomyopathy. Heart Br. Card. Soc..

[24] Chan R.H., Maron B.J., Olivotto I., Pencina M.J., Assenza G.E., Haas T., Lesser J.R., Gruner C., Crean A.M., Rakowski H. (2014). Prognostic value of quantitative contrast-enhanced cardiovascular magnetic resonance for the evaluation of sudden death risk in patients with hypertrophic cardiomyopathy. Circulation.

[25] Adabag A.S., Maron B.J., Appelbaum E., Harrigan C.J., Buros J.L., Gibson C.M., Lesser J.R., Hanna C.A., Udelson J.E., Manning W.J. (2008). Occurrence and frequency of arrhythmias in hypertrophic cardiomyopathy in relation to delayed enhancement on cardiovascular magnetic resonance. J. Am. Coll. Cardiol..

[26] Rudolph A., Abdel-Aty H., Bohl S., Boyé P., Zagrosek A., Dietz R., Schulz-Menger J. (2009). Noninvasive detection of fibrosis applying contrast-enhanced cardiac magnetic resonance in different forms of left ventricular hypertrophy relation to remodeling. J. Am. Coll. Cardiol..

[27] Yang K., Song Y.Y., Chen X.Y., Wang J.X., Li L., Yin G., Zheng Y.C., Wei M.D., Lu M.J., Zhao S.H. (2020). Apical hypertrophic cardiomyopathy with left ventricular apical aneurysm: Prevalence, cardiac magnetic resonance characteristics, and prognosis. Eur. Heart J. Cardiovasc. Imaging.

[28] Moon J.C., McKenna W.J., McCrohon J.A., Elliott P.M., Smith G.C., Pennell D.J. (2003). Toward clinical risk assessment in hypertrophic cardiomyopathy with gadolinium cardiovascular magnetic resonance. J. Am. Coll. Cardiol..

[29] Moon J.C., Reed E., Sheppard M.N., Elkington A.G., Ho S.Y., Burke M., Petrou M., Pennell D.J. (2004). The histologic basis of late gadolinium enhancement cardiovascular magnetic resonance in hypertrophic cardiomyopathy. J. Am. Coll. Cardiol..

[30] Huang L., Ran L., Zhao P., Tang D., Han R., Ai T., Xia L., Tao Q. (2019). MRI native T1 and T2 mapping of myocardial segments in hypertrophic cardiomyopathy: Tissue remodeling manifested prior to structure changes. Br. J. Radiol..

[31] Gastl M., Gotschy A., von Spiczak J., Polacin M., Bönner F., Gruner C., Kelm M., Ruschitzka F., Alkadhi H., Kozerke S. (2019). Cardiovascular magnetic resonance T2* mapping for structural alterations in hypertrophic cardiomyopathy. Eur. J. Radiol. Open.

[32] Snel G., van den Boomen M., Hernandez L.M., Nguyen C.T., Sosnovik D.E., Velthuis B.K., Slart R., Borra R., Prakken N. (2020). Cardiovascular magnetic resonance native T_2_ and T_2_^*^ quantitative values for cardiomyopathies and heart transplantations: A systematic review and meta-analysis. J. Cardiovasc. Magn. Reson. Off. J. Soc. Cardiovasc. Magn. Reson..

[33] Messroghli D.R., Moon J.C., Ferreira V.M., Grosse-Wortmann L., He T., Kellman P., Mascherbauer J., Nezafat R., Salerno M., Schelbert E.B. (2017). Clinical recommendations for cardiovascular magnetic resonance mapping of T1, T2, T2* and extracellular volume: A consensus statement by the Society for Cardiovascular Magnetic Resonance (SCMR) endorsed by the European Association for Cardiovascular Imaging (EACVI). J. Cardiovasc. Magn. Reson. Off. J. Soc. Cardiovasc. Magn. Reson..

[34] Rowin E.J., Maron M.S. (2016). The Role of Cardiac MRI in the Diagnosis and Risk Stratification of Hypertrophic Cardiomyopathy. Arrhythmia Electrophysiol. Rev..

[35] Arcari L., Hinojar R., Engel J., Freiwald T., Platschek S., Zainal H., Zhou H., Vasquez M., Keller T., Rolf A. (2020). Native T1 and T2 provide distinctive signatures in hypertrophic cardiac conditions—Comparison of uremic, hypertensive and hypertrophic cardiomyopathy. Int. J. Cardiol..

[36] Kaldoudi E., Williams C.R. (1993). Relaxation Time Measurements in NMR Imaging. Concepts Magn. Reson..

[37] Amano Y., Takayama M., Kumita S. (2009). Contrast-enhanced myocardial T1-weighted scout (Look-Locker) imaging for the detection of myocardial damages in hypertrophic cardiomyopathy. J. Magn. Reson. Imaging JMRI.

[38] Hurtado-de-Mendoza D., Corona-Villalobos C.P., Pozios I., Gonzales J., Soleimanifard Y., Sivalokanathan S., Montoya-Cerrillo D., Vakrou S., Kamel I., Mormontoy-Laurel W. (2017). Diffuse interstitial fibrosis assessed by cardiac magnetic resonance is associated with dispersion of ventricular repolarization in patients with hypertrophic cardiomyopathy. J. Arrhythmia.

[39] El-Rewaidy H., Neisius U., Nakamori S., Ngo L., Rodriguez J., Manning W.J., Nezafat R. (2020). Characterization of interstitial diffuse fibrosis patterns using texture analysis of myocardial native T1 mapping. PLoS ONE.

[40] Xu J., Zhuang B., Sirajuddin A., Li S., Huang J., Yin G., Song L., Jiang Y., Zhao S., Lu M. (2020). MRI T1 Mapping in Hypertrophic Cardiomyopathy: Evaluation in Patients Without Late Gadolinium Enhancement and Hemodynamic Obstruction. Radiology.

[41] Martinez-Naharro A., Treibel T.A., Abdel-Gadir A., Bulluck H., Zumbo G., Knight D.S., Kotecha T., Francis R., Hutt D.F., Rezk T. (2017). Magnetic Resonance in Transthyretin Cardiac Amyloidosis. J. Am. Coll. Cardiol..

[42] Hinojar R., Varma N., Child N., Goodman B., Jabbour A., Yu C.Y., Gebker R., Doltra A., Kelle S., Khan S. (2015). T1 Mapping in Discrimination of Hypertrophic Phenotypes: Hypertensive Heart Disease and Hypertrophic Cardiomyopathy: Findings from the International T1 Multicenter Cardiovascular Magnetic Resonance Study. Circ. Cardiovasc. Imaging.

[43] Karur G.R., Robison S., Iwanochko R.M., Morel C.F., Crean A.M., Thavendiranathan P., Nguyen E.T., Mathur S., Wasim S., Hanneman K. (2018). Use of Myocardial T1 Mapping at 3.0 T to Differentiate Anderson-Fabry Disease from Hypertrophic Cardiomyopathy. Radiology.

[44] Shi R.Y., An D.A., Chen B.H., Wu R., Du L., Jiang M., Xu J.R., Wu L.M. (2020). Diffusion-weighted imaging in hypertrophic cardiomyopathy: Association with high T2-weighted signal intensity in addition to late gadolinium enhancement. Int. J. Cardiovasc. Imaging.

[45] Gastl M., Lachmann V., Christidi A., Janzarik N., Veulemans V., Haberkorn S., Holzbach L., Jacoby C., Schnackenburg B., Berrisch-Rahmel S. (2021). Cardiac magnetic resonance T2 mapping and feature tracking in athlete’s heart and HCM. Eur. Radiol..

[46] Abdel-Aty H., Cocker M., Strohm O., Filipchuk N., Friedrich M.G. (2008). Abnormalities in T2-weighted cardiovascular magnetic resonance images of hypertrophic cardiomyopathy: Regional distribution and relation to late gadolinium enhancement and severity of hypertrophy. J. Magn. Reson. Imaging JMRI.

[47] Abdel-Aty H., Zagrosek A., Schulz-Menger J., Taylor A.J., Messroghli D., Kumar A., Gross M., Dietz R., Friedrich M.G. (2004). Delayed enhancement and T2-weighted cardiovascular magnetic resonance imaging differentiate acute from chronic myocardial infarction. Circulation.

[48] Gastl M., Gruner C., Labucay K., Gotschy A., Von Spiczak J., Polacin M., Boenner F., Kelm M., Ruschitzka F., Alkadhi H. (2020). Cardiovascular magnetic resonance T2* mapping for the assessment of cardiovascular events in hypertrophic cardiomyopathy. Open Heart.

[49] Cannon C.P. (2008). Lamb BPPHJ: Assessment of Diastolic Function by Cardiac MRI.

[50] Giusca S., Steen H., Montenbruck M., Patel A.R., Pieske B., Erley J., Kelle S., Korosoglou G. (2021). Multi-parametric assessment of left ventricular hypertrophy using late gadolinium enhancement, T1 mapping and strain-encoded cardiovascular magnetic resonance. J. Cardiovasc. Magn. Reson. Off. J. Soc. Cardiovasc. Magn. Reson..

[51] Pagourelias E.D., Mirea O., Duchenne J., Unlu S., Van Cleemput J., Papadopoulos C.E., Bogaert J., Vassilikos V.P., Voigt J.U. (2020). Speckle tracking deformation imaging to detect regional fibrosis in hypertrophic cardiomyopathy: A comparison between 2D and 3D echo modalities. Eur. Heart J. Cardiovasc. Imaging.

[52] Tayal B., Malahfji M., Buergler J.M., Shah D.J., Nagueh S.F. (2021). Hemodynamic determinants of left atrial strain in patients with hypertrophic cardiomyopathy: A combined echocardiography and CMR study. PLoS ONE.

[53] Li Z.L., He S., Xia C.C., Peng W.L., Li L., Liu K.L., Zhang J.G., Pu J., Guo Y.K. (2021). Global longitudinal diastolic strain rate as a novel marker for predicting adverse outcomes in hypertrophic cardiomyopathy by cardiac magnetic resonance tissue tracking. Clin. Radiol..

[54] Cavus E., Muellerleile K., Schellert S., Schneider J., Tahir E., Chevalier C., Jahnke C., Radunski U.K., Adam G., Kirchhof P. (2021). CMR feature tracking strain patterns and their association with circulating cardiac biomarkers in patients with hypertrophic cardiomyopathy. Clin. Res. Cardiol. Off. J. Ger. Card. Soc..

[55] Sivalokanathan S., Zghaib T., Greenland G.V., Vasquez N., Kudchadkar S.M., Kontari E., Lu D.Y., Dolores-Cerna K., van der Geest R.J., Kamel I.R. (2019). Hypertrophic Cardiomyopathy Patients with Paroxysmal Atrial Fibrillation Have a High Burden of Left Atrial Fibrosis by Cardiac Magnetic Resonance Imaging. JACC Clin. Electrophysiol..

[56] Siontis K.C., Geske J.B., Ong K., Nishimura R.A., Ommen S.R., Gersh B.J. (2014). Atrial fibrillation in hypertrophic cardiomyopathy: Prevalence, clinical correlations, and mortality in a large high-risk population. J. Am. Heart Assoc..

[57] Kowallick J.T., Kutty S., Edelmann F., Chiribiri A., Villa A., Steinmetz M., Sohns J.M., Staab W., Bettencourt N., Unterberg-Buchwald C. (2014). Quantification of left atrial strain and strain rate using Cardiovascular Magnetic Resonance myocardial feature tracking: A feasibility study. J. Cardiovasc. Magn. Reson. Off. J. Soc. Cardiovasc. Magn. Reson..

[58] Raman B., Smillie R.W., Mahmod M., Chan K., Ariga R., Nikolaidou C., Ormondroyd E., Thomson K., Harper A.R., Tan G. (2021). Incremental value of left atrial booster and reservoir strain in predicting atrial fibrillation in patients with hypertrophic cardiomyopathy: A cardiovascular magnetic resonance study. J. Cardiovasc. Magn. Reson. Off. J. Soc. Cardiovasc. Magn. Reson..

[59] Pedrizzetti G., Claus P., Kilner P.J., Nagel E. (2016). Principles of cardiovascular magnetic resonance feature tracking and echocardiographic speckle tracking for informed clinical use. J. Cardiovasc. Magn. Reson. Off. J. Soc. Cardiovasc. Magn. Reson..

[60] Ismail T.F., Hsu L.Y., Greve A.M., Gonçalves C., Jabbour A., Gulati A., Hewins B., Mistry N., Wage R., Roughton M. (2014). Coronary microvascular ischemia in hypertrophic cardiomyopathy—A pixel-wise quantitative cardiovascular magnetic resonance perfusion study. J. Cardiovasc. Magn. Reson. Off. J. Soc. Cardiovasc. Magn. Reson..

[61] Varnava A.M., Elliott P.M., Sharma S., McKenna W.J., Davies M.J. (2000). Hypertrophic cardiomyopathy: The interrelation of disarray, fibrosis, and small vessel disease. Heart Br. Card. Soc..

[62] Kim E.K., Lee S.C., Chang S.A., Jang S.Y., Kim S.M., Park S.J., Choi J.O., Park S.W., Jeon E.S., Choe Y.H. (2020). Prevalence and clinical significance of cardiovascular magnetic resonance adenosine stress-induced myocardial perfusion defect in hypertrophic cardiomyopathy. J. Cardiovasc. Magn. Reson. Off. J. Soc. Cardiovasc. Magn. Reson..

[63] Basso C., Thiene G., Corrado D., Buja G., Melacini P., Nava A. (2000). Hypertrophic cardiomyopathy and sudden death in the young: Pathologic evidence of myocardial ischemia. Hum. Pathol..

[64] Maron M.S., Olivotto I., Maron B.J., Prasad S.K., Cecchi F., Udelson J.E., Camici P.G. (2009). The case for myocardial ischemia in hypertrophic cardiomyopathy. J. Am. Coll. Cardiol..

[65] Olivotto I., Girolami F., Sciagrà R., Ackerman M.J., Sotgia B., Bos J.M., Nistri S., Sgalambro A., Grifoni C., Torricelli F. (2011). Microvascular function is selectively impaired in patients with hypertrophic cardiomyopathy and sarcomere myofilament gene mutations. J. Am. Coll. Cardiol..

[66] Jablonowski R., Fernlund E., Aletras A.H., Engblom H., Heiberg E., Liuba P., Arheden H., Carlsson M. (2015). Regional Stress-Induced Ischemia in Non-fibrotic Hypertrophied Myocardium in Young HCM Patients. Pediatric Cardiol..

[67] Chiribiri A., Leuzzi S., Conte M.R., Bongioanni S., Bratis K., Olivotti L., De Rosa C., Lardone E., Di Donna P., Villa A.D. (2015). Rest perfusion abnormalities in hypertrophic cardiomyopathy: Correlation with myocardial fibrosis and risk factors for sudden cardiac death. Clin. Radiol..

[68] Raman B., Ariga R., Spartera M., Sivalokanathan S., Chan K., Dass S., Petersen S.E., Daniels M.J., Francis J., Smillie R. (2019). Progression of myocardial fibrosis in hypertrophic cardiomyopathy: Mechanisms and clinical implications. Eur. Heart J. Cardiovasc. Imaging.

[69] Tezuka D., Kosuge H., Terashima M., Koyama N., Kishida T., Tada Y., Suzuki J.I., Sasano T., Ashikaga T., Hirao K. (2018). Myocardial perfusion reserve quantified by cardiac magnetic resonance imaging is associated with late gadolinium enhancement in hypertrophic cardiomyopathy. Heart Vessel..

[70] Knaapen P., van Dockum W.G., Götte M.J., Broeze K.A., Kuijer J.P., Zwanenburg J.J., Marcus J.T., Kok W.E., van Rossum A.C., Lammertsma A.A. (2006). Regional heterogeneity of resting perfusion in hypertrophic cardiomyopathy is related to delayed contrast enhancement but not to systolic function: A PET and MRI study. J. Nucl. Cardiol. Off. Publ. Am. Soc. Nucl. Cardiol..

[71] Petersen S.E., Jerosch-Herold M., Hudsmith L.E., Robson M.D., Francis J.M., Doll H.A., Selvanayagam J.B., Neubauer S., Watkins H. (2007). Evidence for microvascular dysfunction in hypertrophic cardiomyopathy: New insights from multiparametric magnetic resonance imaging. Circulation.

[72] Hughes R.K., Camaioni C., Augusto J.B., Knott K., Quinn E., Captur G., Seraphim A., Joy G., Syrris P., Elliott P.M. (2021). Myocardial Perfusion Defects in Hypertrophic Cardiomyopathy Mutation Carriers. J. Am. Heart Assoc..

[73] Sipola P., Lauerma K., Husso-Saastamoinen M., Kuikka J.T., Vanninen E., Laitinen T., Manninen H., Niemi P., Peuhkurinen K., Jääskeläinen P. (2003). First-pass MR imaging in the assessment of perfusion impairment in patients with hypertrophic cardiomyopathy and the Asp175Asn mutation of the alpha-tropomyosin gene. Radiology.

[74] Das A., Kelly C., Teh I., Nguyen C., Brown L., Chowdhary A., Jex N., Thirunavukarasu S., Sharrack N., Gorecka M. (2021). Phenotyping hypertrophic cardiomyopathy using cardiac diffusion magnetic resonance imaging: The relationship between microvascular dysfunction and microstructural changes. Eur. Heart J. Cardiovasc. Imaging.

[75] McGill L.A., Ismail T.F., Nielles-Vallespin S., Ferreira P., Scott A.D., Roughton M., Kilner P.J., Ho S.Y., McCarthy K.P., Gatehouse P.D. (2012). Reproducibility of in-vivo diffusion tensor cardiovascular magnetic resonance in hypertrophic cardiomyopathy. J. Cardiovasc. Magn. Reson. Off. J. Soc. Cardiovasc. Magn. Reson..

[76] Ariga R., Tunnicliffe E.M., Manohar S.G., Mahmod M., Raman B., Piechnik S.K., Francis J.M., Robson M.D., Neubauer S., Watkins H. (2019). Identification of Myocardial Disarray in Patients with Hypertrophic Cardiomyopathy and Ventricular Arrhythmias. J. Am. Coll. Cardiol..

[77] Nielles-Vallespin S., Khalique Z., Ferreira P.F., de Silva R., Scott A.D., Kilner P., McGill L.A., Giannakidis A., Gatehouse P.D., Ennis D. (2017). Assessment of Myocardial Microstructural Dynamics by In Vivo Diffusion Tensor Cardiac Magnetic Resonance. J. Am. Coll. Cardiol..

[78] Wymer D.T., Patel K.P., Burke W.F., Bhatia V.K. (2020). Phase-Contrast MRI: Physics, Techniques, and Clinical Applications. Radiographics.

[79] Srichai M.B., Lim R.P., Wong S., Lee V.S. (2009). Cardiovascular applications of phase-contrast MRI. AJR Am. J. Roentgenol..

[80] Nayak K.S., Nielsen J.F., Bernstein M.A., Markl M., DGatehouse P., MBotnar R., Saloner D., Lorenz C., Wen H., SHu B. (2015). Cardiovascular magnetic resonance phase contrast imaging. J. Cardiovasc. Magn. Reson. Off. J. Soc. Cardiovasc. Magn. Reson..

[81] Stankovic Z., Allen B.D., Garcia J., Jarvis K.B., Markl M. (2014). 4D flow imaging with MRI. Cardiovasc. Diagn. Ther..

[82] Pruijssen J.T., Allen B.D., Barker A.J., Bonow R.O., Choudhury L., Carr J.C., Markl M., van Ooij P. (2020). Hypertrophic Cardiomyopathy Is Associated with Altered Left Ventricular 3D Blood Flow Dynamics. Radiology. Cardiothorac. Imaging.

[83] She J.Q., Guo J.J., Yu Y.F., Zhao S.H., Chen Y.Y., Ge M.Y., Zeng M.S., Jin H. (2021). Left Ventricular Outflow Tract Obstruction in Hypertrophic Cardiomyopathy: The Utility of Myocardial Strain Based on Cardiac MR Tissue Tracking. J. Magn. Reson. Imaging: JMRI.

[84] Van Ooij P., Allen B.D., Contaldi C., Garcia J., Collins J., Carr J., Choudhury L., Bonow R.O., Barker A.J., Markl M. (2016). 4D flow MRI and T1 -Mapping: Assessment of altered cardiac hemodynamics and extracellular volume fraction in hypertrophic cardiomyopathy. J. Magn. Reson. Imaging: JMRI.

[85] Marstrand P., Han L., Day S.M., Olivotto I., Ashley E.A., Michels M., Pereira A.C., Wittekind S.G., Helms A., Saberi S. (2020). Hypertrophic Cardiomyopathy with Left Ventricular Systolic Dysfunction: Insights from the SHaRe Registry. Circulation.

[86] Wang Z.J., Ohliger M.A., Larson P., Gordon J.W., Bok R.A., Slater J., Villanueva-Meyer J.E., Hess C.P., Kurhanewicz J., Vigneron D.B. (2019). Hyperpolarized ^13^C MRI: State of the Art and Future Directions. Radiology.

[87] Arenja N., Fritz T., Andre F., Riffel J.H., Aus dem Siepen F., Ochs M., Paffhausen J., Hegenbart U., Schönland S., Müller-Hennessen M. (2017). Myocardial contraction fraction derived from cardiovascular magnetic resonance cine images-reference values and performance in patients with heart failure and left ventricular hypertrophy. Eur. Heart J. Cardiovasc. Imaging.

[88] Page M.J., McKenzie J.E., Bossuyt P.M., Boutron I., Hoffmann T.C., Mulrow C.D., Shamseer L., Tetzlaff J.M., Akl E.A., Brennan S.E. (2021). The PRISMA 2020 statement: An updated guideline for reporting systematic reviews. BMJ.

